# Method of Treating Intermittent Fevers

**Published:** 1803-04-01

**Authors:** 

**Affiliations:** Bamberg


### Method *of treating* Intermittent Fevers; *by* Dr. Marcus, *of Bamberg*.

Intermittent Fevers seem principally to originate in direct asthenia, and the chief indication to be attended to in treating them, consists in augmenting the inciting powers to such a degree, that a just incitation be produced in the system of organisation. Above all, it is necessary to find out the degree of asthenia, in order to be capable of employing a proper degree of incitement, either by diminishing the incitants accordingly, or by increasing them; a circumstance, which can only be ascertained by the acuteness and judgement of the practitioner. It may be suggested, that in proportion to the greater degree of direct asthenia, the more ought the remedies to be of the volatile and diffusible kind; and the smaller the doses and the shorter the intervals, in which they are to be administered. This rule particularly deserves the attention of the physician. In Quotidians, therefore, more volatile inciting remedies are to be employed, and at the same time in smaller doses and intervals, than in Tertians, and in these again more than in the Quartans. In a high degree of debility, it is advisable to change the inciting remedies from time to time, to combine them in different modes, by which means their effects will be greatly heightened. The principal and most efficacious remedies that may be used in this fever, are opium, musk, camphor, æther vitrioli, alkalis, strong wine, warm baths, warm aromatic fomentations, spirituous frictions, a nourishing diet, meat, eggs, spicy preparations, &c.

In a Quotidian Fever, Dr. M. proceeds in the following manner: In the beginning he gives every hour, or every half half hour, or every quarter of an hour, camphor and tincture of opium alternately, six drops of which contain one grain of opium; he begins with one or two drops in short intervals, raising the dose by degrees to five or six drops. The patient is at the same time put into a warm bath, which is mixed with aromatic herbs; he is rubbed over with spirit of wine, ordered to drink a glass of wine, and to take broth impregnated with spices. When this method is continued for some days, it is necessary to attend to its success, and if the patient should find himself better, the doses of the former remedies must be increased, and the intervals in which they are administered lengthened. It is a very essential point in this method of cure, that the remedies should be continued without interruption day and night, and the principal success, of which we may now boast in removing the fever in so short a time, seems to be owing to this circumstance, which has formerly been almost utterly neglected. It is generally the case that the medicines are discontinued by night, in which way the proper degree of incitation could never be attained, and the *stadium reconvalescentiae* is thus unnecessarily protracted, very much to the disadvantage of the patient. Another material fault in the cure of these fevers, according to the former method, consists in the remedies being discontinued during the paroxysm, or as it were, by too much inciting the patient by large doses, by which the degree of debility must be undoubtedly increased. If however in the above manner the incitation of all the systems of organization be gradually increased, and the due and congenial action of organism begins to be restored, which we perceive from the paroxysms becoming shorter, and more intermitting, &c. then it is advisable to combine the diffusible stimuli with permanent irritants or roborants, particularly in form of *infusum frigidum,* as with tincture of opium or æther vitrioli. About this time we may also allow the use of meat, but at the same time we ought to take care of not admitting the use of watery liquors, lemonade, &c. to which the patients frequently resort, in order to quench the thirst; vegetables and fruits are likewise to be strictly prohibited, by which the patients often experience a return of the fever. As a drink, we recommend a mixture of wine and water, but from time to time to take a glass of pure wine; if the circumstances of the patient should not permit him the use of wine, he may drink a mixture of alcohol with water, or an infusion of *flores arnicae* with liquor. anodyn. Hoffm. and take now and then a glass of any good liquor. In most cases we succeeded in removing intermittent intermittent fevers, in a short time, by only attending to this method of cure, and we were never obliged to resort to any specific remedy. The Peruvian bark is employed as a permanent inciting remedy, when the organs of assimilation have regained their proper action, or when the patient begins to recover; it is then given at first with volatile remedies, and afterwards by itself.

